# Editorial for the *IJMS* Special Issue on “Molecular Genetics of Autism and Intellectual Disability”

**DOI:** 10.3390/ijms241210394

**Published:** 2023-06-20

**Authors:** Hyung-Goo Kim

**Affiliations:** Neurological Disorders Research Center, Qatar Biomedical Research Institute, Hamad Bin Khalifa University, Doha P.O. Box 34110, Qatar; hkim@hbku.edu.qa

Autism spectrum disorder (ASD), a neurodevelopmental illness that affects children at an early age with a global prevalence of 1%, is diagnosed based on clinical features such as social impairment, repetitive behaviors, and restricted interests. ASD is genetically heterogeneous, and the genetic etiology of ASD remains unknown in 20–60% of autistic people. ASD subjects commonly have co-occurring comorbidities such as intellectual disability (ID), seizures, sleep problems, or digestive problems, which suggest more complex genetic etiologies. Exome sequencing and other next-generation sequencing (NGS) techniques have been successful in increasing the total number of known ASD genes.

To advance research in the field of autism, we are pleased to present this Special Issue of the *International Journal of Molecular Sciences*, entitled “Molecular Genetics of Autism and Intellectual Disability”. This Special Issue includes a total of six state-of-the-art, outstanding contributions (four original articles and two reviews) providing new information in the field of autism on associated new candidate genes, as well as the molecular mechanism and salivary biomarkers of autism.

In the first article in this Special Issue (SI) by Collins et al. [1], the authors studied the effect of Chromodomain helicase DNA-binding protein-7 (CHD7) mutations, representing a common cause of the rare congenital condition known as CHARGE syndrome [2]. Although brain-related abnormalities in CHARGE syndrome patients are not used for the diagnosis of this condition, they may exhibit neurodevelopmental disorders such as autism spectrum and hyperactivity disorders. These clinical features are linked to several structural abnormalities in the brain, including hindbrain malformations; malformations in the hippocampus, hypothalamus, and posterior fossa; cortical atrophy; ventricular enlargement; and the dysgenesis of the corpus callosum. The authors generated two Chd7 heterozygous loss-of-function mouse models with different inbred and outbred genetic backgrounds to recapitulate the phenotype of CHARGE syndrome patients, since homozygous mice are embryonic-lethal. The authors found that CHARGE syndrome’s core clinical manifestations were consistent in both mouse models, but the inbred Chd7^+/Whi^ model’s phenotypes were more severe than those of the Chd7^+/tm2a^ mixed-background mice, occasionally showing reduced penetrance. The phenotypes manifested were hyperactivity, growth delays, impaired grip strength, and repetitive behaviors, in addition to dysgenesis of the corpus callosum and hypoplasia of the hippocampus. The authors concluded that the genetic background and modifiers are most likely responsible for the phenotypic diversity seen in people with CHARGE syndrome. This paper is important for neurodevelopmental disorders such as autism and intellectual disability, because it provides new insights into the characteristics of CHARGE syndrome using two mouse models with Chd7 heterozygous loss-of-function. Specifically, the paper reveals reduced levels of insulin and dysgenesis of the corpus callosum, which have not previously been reported in CHARGE syndrome. This suggests the need for a more careful examination of this newly identified phenotype in CHARGE patients. Agenesis of the corpus callosum has previously been associated with behavioral problems [3]. This information can aid us in understanding and diagnosing these disorders in the future. CHD7 is also involved in Kallmann syndrome, characterized by anosmia and hypogonadotropic hypogonadism, neither of which, unfortunately, has been characterized in these mouse models [4].

A large number of disease genes responsible for intellectual disability (ID) and autism are still unknown, despite the fact that more than 1500 genes are associated with ID and/or autism [5]. As our understanding of genetics and the brain continues to grow, more genes are being identified as potential contributors to autism and intellectual disability. In the next article in this SI, Bruno et al. [6] identified four de novo disruptive mutations in four novel potential ASD/ID genes (*MBP*, *PCDHA1*, *PCDH15*, and *PDPR*). Furthermore, VUSs were identified alone or in combination in 26 novel genes (*RGPD4*, *RIN3*, *SBNO2*, *RBMS1*, *SORBS1*, *CHRFAM7A*, *RSF1*, *AGPAT5*, *GALR3*, *NRXN2*, *TYRO3*, *MAP3K10*, *SLC7A8*, *LONP1*, *CBX3*, *DCAF11*, *GINS2*, *SLC8A1*, *CDC7*, *IPO7*, *POTEH*, *MAP3K5*, *SORBS1*, *POU3F2*, *KANSL3*, *IQSEC3*). The authors also discovered eight pathogenic variants in already known ID/ASD genes (*SYNGAP1*, *SMAD6*, *PACS1*, *SHANK3*, *KMT2A*, *KCNQ2*, *ACTB*, and *POGZ*) and VUSs in ten known ID/ASD genes (*BRWD3*, *CNOT1*, *DNMT3A*, *FGF13*, *HUWE1*, *KMT2B*, *NLGN4X*, *PHF8*, *TAF1*, *DDX3X*) by carrying out the whole-exome sequencing (WES) of 60 trios, the majority of whom had syndromic ID/ASD.

In their contribution, Jagomae et al. [7] delved into the role of the IgLON superfamily of cell adhesion molecules in brain development and their associations with neuropsychiatric disorders. The development of vertebrate brains involves the formation of neural progenitor cells, neuronal differentiation, and axon guidance, which are regulated by cell adhesion molecules such as the IgLON superfamily, including LSAMP, NTM, OPCML, NEGR1, and IgLON5. Several IgLON loci polymorphisms and imbalances in IgLON expression levels are associated with cognitive dysfunction and a wide range of neuropsychiatric disorders in humans, including autism, intellectual disability, schizophrenia, major depression, and bipolar disorder. Genetic deficiencies in IgLONs cause neurodevelopmental and behavioral anomalies in mice, sharing characteristics with neuropsychiatric disorders in humans. While LSAMP, NTM, and OPCML have two alternative promotors, NEGR1 and IgLON5 have a single promotor. The alternative promotor-specific spatio-temporal expression profiles of IgLONs in mouse brains observed in this study suggest the importance of IgLON alternative promoter usage in helping to coordinate the complexly integrated functions of neural cell proliferation, differentiation, and morphogenesis with the more specialized substructures. Investigation of these spatial and temporal expression patterns is crucial for understanding how IgLON genes are implicated in brain development linked to neuropsychiatric disorders.

The article of Win-Shwe et al. [8] discusses the potential association between exposure to air pollutants and the development of neurodevelopmental and neurodegenerative disorders such as ASD and Alzheimer’s disease. Human studies have found that maternal exposure to air pollution particulate matter (PM_2.5_) during pregnancy is associated with a greater risk of ASD in children, while animal studies have shown that exposure to ultrafine ambient particles during the early postnatal period induces ASD-like behavior and neuroinflammation in mice. A recent study examined the effects of perinatal exposure to diesel-exhaust-origin secondary aerosol (DE-SOA) on autism-like behavior and neuroimmune responses in rats, finding that DE-SOA exposure induced autism-like behavior and neuroinflammation, affecting the rats’ social behavior and causing abnormal N-methyl-D-aspartate (NMDA) receptor expression in the hippocampus. Additionally, exposure to DE-SOA was associated with a decrease in maternal performance, accompanied by a decrease in estrogen receptor (ER)-alpha and oxytocin receptor expression in female mice. The imbalance in glutamate/GABA was proposed as a potential mechanism of ASD. These findings suggest that exposure to air pollutants during the gestational and neonatal periods may increase the risk of developing ASD, and more research is needed to understand the precise etiology and pathophysiology of autism. The results of this study have significant implications for pregnant women and developing fetuses, as they provide insight into potential neurodevelopmental disorders associated with exposure to air pollution.

The review of Wang et al. [9] discusses the challenges of studying ASD, a complex disorder with multiple risk factors and no physical or pharmacological treatment. Diagnosis typically occurs later in life and requires multidisciplinary assessment and targeted interventions, which can reduce some symptoms but not cure the disorder. Recent advances in ASD research have used transgenic mouse models and altered environmental factors to investigate neuropathology, identifying hundreds of genes as risk factors for ASD development. Gene therapy has emerged as a potential personalized treatment strategy. However, mechanistic treatments will only succeed when the heterogeneity of neurodevelopmental disorders is incorporated into precision medicine through multidisciplinary integration. Attention to potential links between biochemical molecular systems, neural circuits, and environmental variables is necessary to optimize therapeutic approaches for ASD. The study of ASD should also consider potential biomarkers and comorbidities, as multiple clinical disorders frequently coexist in autism. Large-scale population-based cohort studies are necessary to identify dynamic spatial and temporal links between behavior, development, and comorbidity types. While the current treatments, including drugs and non-drugs, relieve some symptoms, in-depth study of the disease’s core defects and the normalization of its pathophysiology are required to develop effective therapies.

Researchers are searching for biomarkers in blood, amniotic fluid, and saliva for various purposes. Saliva is often overlooked, but it has many advantages, including detectable markers comparable to those found in blood, making it a useful alternative. Janšáková et al. [10] aimed to evaluate the potential of using salivary biomarkers in autism research. They conducted a systematic review of studies published between 2010 and 2020 that investigated salivary biomarkers in individuals with ASD. The review found that salivary biomarkers have the potential to aid in the diagnosis and monitoring of ASD. Several studies demonstrated significant differences in salivary biomarkers between individuals with ASD and neurotypical controls. Specifically, changes in the salivary levels of cortisol, melatonin, oxytocin, and cytokines were observed in individuals with ASD. Additionally, salivary biomarkers may be useful for predicting treatment response and identifying subgroups of individuals with ASD. This review also identified some limitations of using salivary biomarkers in ASD research. Variability in sample collection methods and timing, as well as the presence of confounding factors such as age, medication use, and comorbidities, can impact the accuracy and reliability of salivary biomarker measurements. However, the review also highlights the potential of using salivary biomarkers in combination with other diagnostic tools to improve the accuracy of ASD diagnosis and personalized treatment. Overall, the review suggests that salivary biomarkers hold promise as a non-invasive and cost-effective tool for ASD research, but further studies are needed to establish their clinical utility and standardize their measurement protocols.

The six papers in this Special Issue demonstrate recent advances in autism research, covering a wide range of topics and providing important insights into the biological mechanism, neurodevelopmental risk factors, precision therapy, and salivary biomarkers for the diagnosis of autism. This research highlights the importance of interdisciplinary collaboration in understanding the complex genetic, molecular, and environmental factors contributing to neurodevelopmental disorders and provides promising avenues for further research and potential therapeutic targets. This Special Issue is an important reference for scientists studying autism and is intended to inspire more researchers to focus on the study of the molecular genetics of autism and intellectual disability.

The author expresses his gratitude to the *International Journal of Molecular Sciences* and the reviewers for their support and insightful contributions to this Special Issue. It is anticipated that this Special Issue will garner a great deal of interest and add to the body of knowledge on the molecular genetics of autism and intellectual disability.
